# Refining eligibility criteria of unit selection for myeloablative cord blood transplantation in acute leukemia: Real‐world experience of a referral center

**DOI:** 10.1002/jha2.703

**Published:** 2023-04-25

**Authors:** Zimin Sun, Yu Hu, Yanping Ji, Xueou Liu, Xiaowen Gong, Yahui Feng, Huilan Liu, Wei Zhang, Saibing Qi, Qiujin Shen, Kaidi Song, Liangquan Geng, Wen Yao, Xiang Wan, Baolin Tang, Xiaoyu Zhu, Guangyu Sun, Ping Qiang, Zhen Song, Junren Chen

**Affiliations:** ^1^ Department of Hematology The First Affiliated Hospital of University of Science and Technology of China Hefei China; ^2^ Blood and Cell Therapy Institute Division of Life Sciences and Medicine Anhui Provincial Key Laboratory of Blood Research and Applications University of Science and Technology of China Hefei China; ^3^ State Key Laboratory of Experimental Hematology National Clinical Research Center for Blood Diseases Haihe Laboratory of Cell Ecosystem Institute of Hematology and Blood Diseases Hospital Chinese Academy of Medical Sciences and Peking Union Medical College Tianjin China; ^4^ Tianjin Institutes of Health Science Tianjin China; ^5^ School of Clinical Medicine Anhui Medical University Hefei China; ^6^ Department of Hematology Affiliated Hospital of Jiangsu University Zhenjiang China

**Keywords:** alternative donors, umbilical cord blood transplantation

## Abstract

The algorithm for cord blood (CB) unit selection is still somewhat ambiguous. We retrospectively analyzed 620 cases of acute leukemia between 2015 and 2020, who were treated with myeloablative single‐unit umbilical CB transplantation (UCBT). We found that, when human leukocyte antigen (HLA) mismatch was ≤3/10, CD34^+^ cell dosage <0.83 × 10^5^/kg—considerably lower than prevalent guidelines—was permissible without affecting survival. Moreover, synergy between donor killer‐cell immunoglobulin‐like receptors (KIR) haplotypes‐B and donor‐recipient HLA‐C mismatch protected against relapse‐related mortality. We submit that minimum required CD34^+^ cell dosage can possibly be relaxed to broaden access to UCBT, and donor KIR genotyping should be considered during unit selection.

## 1 INTRODUCTION

Cord blood (CB) is an important alternative source of allogeneic transplants, especially if we consider its shorter wait time, superior anti‐leukemia effect [1, 2], and comparatively faster long‐term immune reconstitution [3]. Donor selection for umbilical CB transplantation (UCBT) needs to consider multiple factors [4], and a few issues still remain:

First, what is the minimum required CD34^+^ cell dosage? Earlier research proposed a minimum CD34^+^ cell dosage of 1.7 × 10^5^/kg [5]. More recent data of acute myeloid leukemia (AML) in complete remission suggested that much lower cell dosage might be permissible [6].

Second, what is the optimal trade‐off between CD34^+^ cell dosage and human leukocyte antigen (HLA) match? Previous researchers reported that higher cell count could compensate for higher HLA mismatch in UCBT [7]; this observation, however, was based on total nucleated cell dosage, not CD34^+^ cell dosage, which better correlates with hematopoietic recovery [8]. Moreover, although earlier research suggested that even mismatch at one HLA allele affected non‐relapse mortality (NRM) in UCBT [9], more recent data suggested that as much as 4/8 mismatch might be permissible [10].

Third, is there a role for killer‐cell immunoglobulin‐like receptors (KIR)? There has been large‐cohort analysis of the role of KIR ligand mismatch (which only requires HLA typing) [11] but no published dataset related to donor KIR genotype (which requires genotyping of the KIR genes) in UCBT [12]. In non‐CB HSCT, we know that donor‐recipient HLA‐C mismatch can enhance the protective effect of donor KIR2DS1 against leukemia relapse [13], but this hypothesis has never been tested in UCBT.

To sum up, the algorithm for unit selection is still somewhat ambiguous for practitioners.

## 2 METHODS

We conducted a retrospective analysis of 620 cases (age 0.7–62.3) of acute leukemia between 2015 and 2020, who were treated with unrelated single‐unit UCBT with myeloablative conditioning (MAC) without antithymocyte globulin at the First Affiliated Hospital of University of Science and Technology of China (FAHUSTC) (Table S1, Figure S1 and Supplementary Methods).

“Intermediate‐risk AML” and “adverse‐risk AML” were defined according to the 2022 European LeukemiaNet (ELN) risk classification by genetics at initial diagnosis [14]. “Poor‐risk acute lymphoblastic leukemia (ALL)” was defined as presenting complex karyotype, hypoploidy, BCR‐ABL1 fusion, TCF3‐HLF fusion, MLL rearrangement, t(v; 14q32)/IgH, IKZF1 alteration, TP53 mutation, alterations associated with Ph‐like ALL (for example, mutations in JAK1/2/3 or FLT3), or T‐lineage phenotype at initial diagnosis [15]. (Abbreviations: BCR, breakpoint cluster region; ABL1, Abelson murine leukemia viral oncogene homolog 1; TCF3, transcription factor 3; HLF, hepatic leukemia factor; MLL, mixed‐lineage leukemia; IgH, immunoglobulin heavy chain; IKZF1, Ikaros family zinc finger 1; TP53, tumor protein P53; Ph, Philadelphia chromosome; JAK, Janus kinase; FLT3, Feline McDonough Sarcoma‐like tyrosine kinase 3.)

All the CB donors were genotyped for the presence/absence of KIR genes (KIR2DL1, KIR2DL2, KIR2DL3, KIR2DL4, KIR2DL5, KIR2DS1, KIR3DS1, KIR2DS2, KIR2DS3, KIR2DS4, KIR2DS5, KIR3DL1, KIR3DL2, KIR3DL3, KIR3DP1). Donor and recipient genotypes at the HLA‐C locus were classified into types “C1” and “C2” based on allele groups (Supplementary Methods). Inhibitory KIR ligand mismatch was defined in the graft‐versus‐host direction (For instance, C1 ligand graft‐versus‐host mismatch was defined as when a type C1 ligand was present in the donor but not in the recipient.)

We conducted multivariate analyses to investigate how CD34^+^ cell dosage, HLA mismatch, inhibitory KIR mismatch, and donor KIR haplotype collectively influenced relapse, relapse‐related mortality (RRM), NRM, overall survival (OS), and neutrophil engraftment (NE) after correcting for covariates such as year, sex, age, primary disease, disease risk according to genetics, and disease status at transplant. (All the cases were MAC.) For interactions between donor KIR and HLA, we asked if type C1 and C2 ligand graft‐versus‐host mismatch modulated the effect of donor KIR haplotypes‐B. Relapse and NRM were treated as mutual competing risks for each other. NRM was treated as a competing risk for RRM. All‐cause death was treated as a competing risk for NE. Proportional hazards models for subdistributions of competing risks were computed with the Fine‐Gray method, while cumulative incidence curves were compared with the Gray method. All the reported *p* values were not adjusted for multiple‐hypothesis testing.

Multivariate analyses were also conducted in subgroups (children, adults, ALL, or AML).

## 3 RESULTS

**FIGURE 1:**
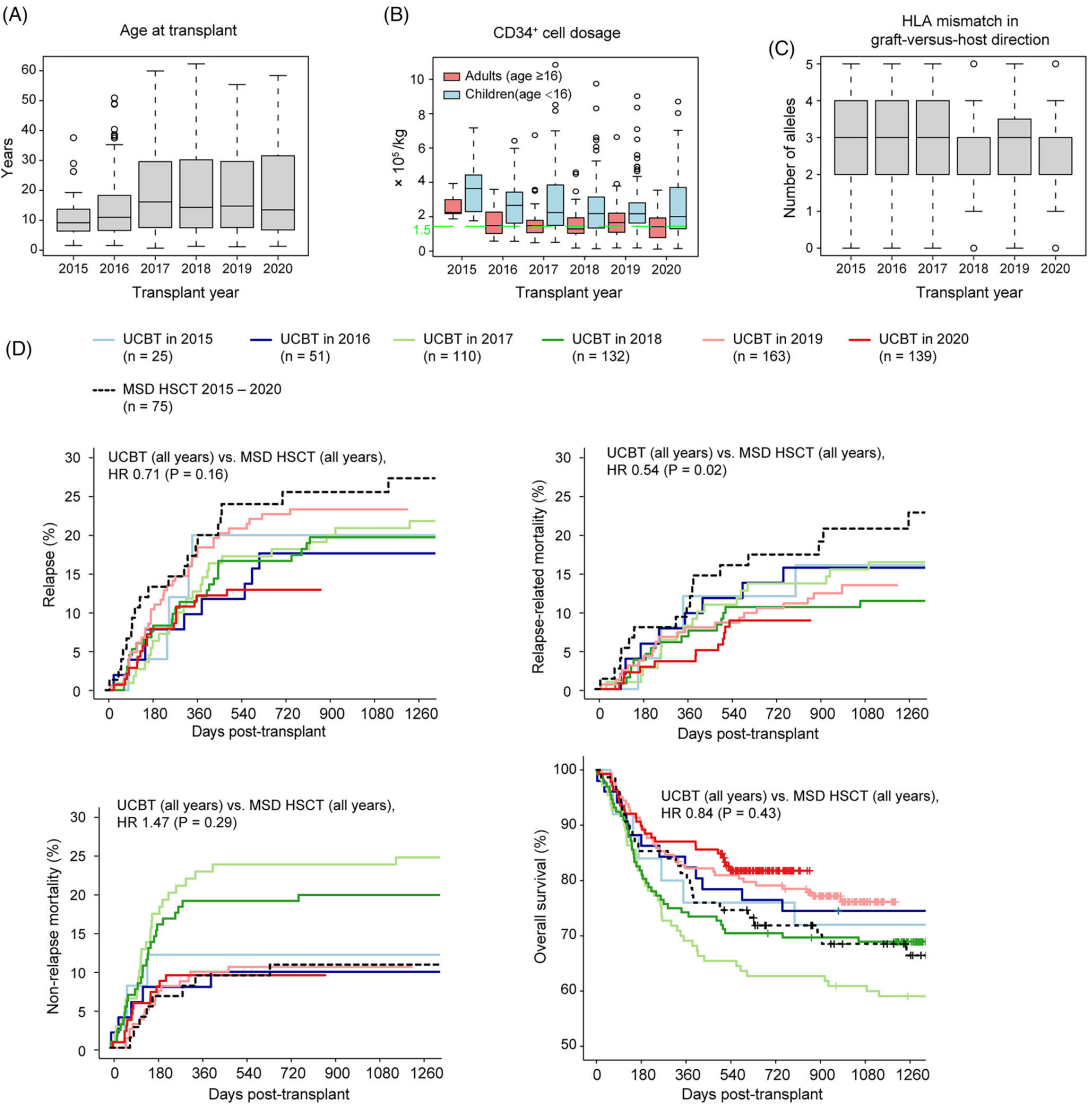
Temporal evolution of umbilical cord blood transplantation (UCBT) characteristics in the study cohort. (A) Patient age. (B) CD34^+^ cell dosage. (C) Human leukocyte antigen (HLA) mismatch in graft‐versus‐host direction. (D) Clinical outcomes (relapse, relapse‐related mortality, nonrelapse mortality, and overall survival). Matched sibling donor (MSD) HSCT cases are also plotted for comparison.

270 (44%), 340 (55%), and 10 (2%) of the cases were ALL, AML, and mixed‐phenotype acute leukemia, respectively. 353 cases (57%) had achieved the first complete remission (CR1) before transplantation. During the search for CB units, we tried to ensure HLA match ≥4/6, ≥5/8, or ≥6/10, CD34^+^ cell dosage ≥1 × 10^5^/kg, and no detectable donor‐specific antibody in the recipients; 114 cases (18%), however, did not meet all the criteria (Table S1 and Figure S2). Over time, there were steady trends of increasing patient age (Figure 1A), decreasing CD34^+^ cell dosage (Figure 1B), and better HLA match (Figure 1C). Indeed, between 2015 and 2020, our percentage of cases with CD34^+^ cell dosage <1.5 × 10^5^/kg increased from 0% to 38%, and as a consequence the percentage of treated adults increased from 20% to 43%. The 5th‐ and 10th‐percentile values of CD34^+^ cell dosage in the study cohort were 0.60 and 0.83 × 10^5^/kg, respectively.

The median follow‐up duration was 931 days (range, 2–2636). Relapse, RRM, NRM, and OS were 18%, 11%, 14%, and 75% at 2 years posttransplant, respectively (Supplementary Table S1). For comparison, during the study period, there were 75 additional cases of acute leukemia that were treated with myeloablative matched sibling donors (MSD) HSCT at the FAHUSTC (Table S1 and Figure S3). The 620 UCBT cases had statistically indistinguishable relapse incidence (hazard ratio [HR] 0.71; 95% confidence interval [CI] 0.44–1.14; *p* = 0.16), NRM (HR 1.47; 95% CI 0.72–3.00; *p* = 0.29), and OS (HR 0.84; 95% CI 0.56–1.29; *p* = 0.43) compared to the MSD HSCT cases (Figure 1D). UCBT, however, had significantly lower relapse‐related mortality (RRM; HR 0.54; 95% CI 0.32–0.91; *p* = 0.021) than MSD HSCT (Figure 1D).

In our Han ethnic‐predominant study cohort, KIR2DL2 and KIR2DS2—commonly associated with “centromeric haplotype‐B” (CenB)—were in tight linkage disequilibrium (LD; *r* = 0.96), while KIR2DS1 and KIR3DS1—both associated with “telomeric haplotype‐B” (TelB)—and KIR2DL5 were in strong mutual LD (*r* ≥0.83) (Figure S4). CenB and TelB were present in 144 (23%) and 263 (42%) of the donors, respectively. ≈61% and ≈34% of the study cohort were C1/C1 and C1/C2, respectively, at the HLA‐C locus. One hundred nineteen (19%) of the cases had inhibitory KIR ligand mismatch in the graft‐versus‐host direction (that is, an inhibitory ligand was present in the donor but not the recipient).

**FIGURE 2:**
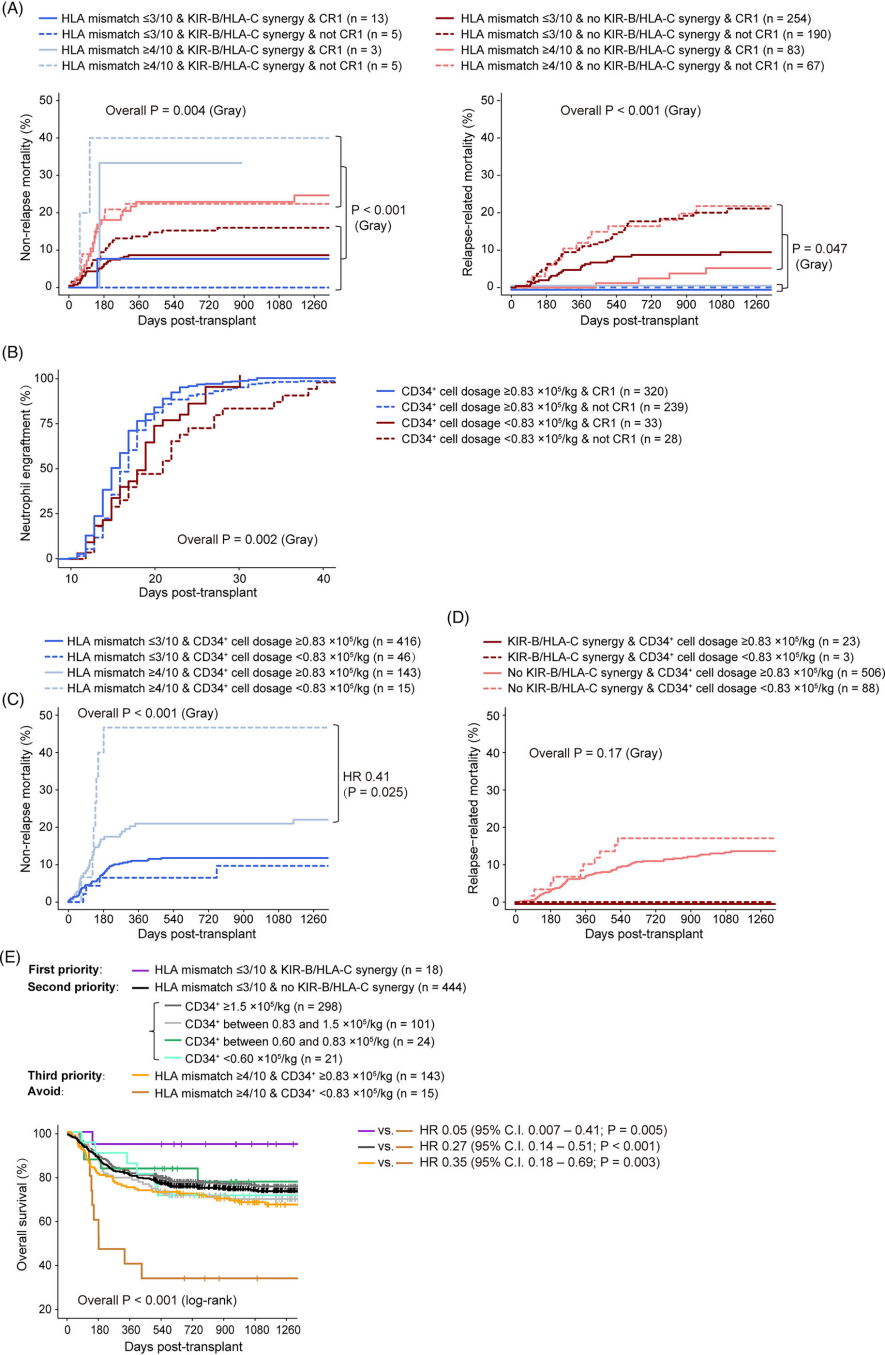
Impact of human leukocyte antigen (HLA), killer‐cell immunoglobulin‐like receptors (KIR), CD34^+^ cell dosage, and disease status on clinical outcomes of umbilical cord blood transplantation (UCBT). (A) Impact of HLA, KIR, and disease status on nonrelapse mortality and relapse‐related mortality. “KIR‐B/HLA‐C synergy” was defined as “donor CenB and type C1 ligand graft‐versus‐host mismatch,” “donor TelB and type C1 ligand graft‐versus‐host mismatch,” or “donor TelB and type C2 ligand graft‐versus‐host mismatch.” (B) Impact of CD34^+^ cell dosage and disease status at transplant on neutrophil engraftment. (C) Nonrelapse mortality conditional on CD34^+^ cell dosage and HLA mismatch. (D) Relapse‐related mortality conditional on CD34^+^ cell dosage and presence/absence of “KIR‐B/HLA‐C synergy.” (E) Proposed priority rules for unit selection.

Multivariate analysis (Supplementary Table S2) indicated that CR1 (HR 0.35; 95% CI 0.24–0.52; *p* < 0.001) was the most predictive factor for relapse. Both CR1 (HR 0.33; 95% CI 0.20–0.53; *p* < 0.001) and a set of combinations of donor KIR haplotype‐B and donor‐recipient ligand mismatch (“donor CenB and type C1 ligand graft‐versus‐host mismatch,” “donor TelB and type C1 ligand graft‐versus‐host mismatch,” and “donor TelB and type C2 ligand graft‐versus‐host mismatch”—collectively called “KIR‐B/HLA‐C synergy” here; HR < 0.01; 95% CI < 0.01; *p* < 0.001) were significantly associated with lower RRM. (None of the “KIR‐B/HLA‐C synergistic” cases [*n* = 26] had RRM events.) HLA mismatch ≤3/10 (HR 0.46; 95% CI 0.30–0.72; *p* < 0.001) was significantly associated with lower NRM. Therefore, there were differential effects of HLA match and natural killer alloreactivity on the clinical outcomes (Figure 2A): Cases that had better HLA match (mismatch ≤3/10) tended to have lower NRM (*p* < 0.001), while “KIR‐B/HLA‐C synergistic” cases had the lowest RRM (*p* = 0.047).

Subgroup analyses of children (age < 16; *n* = 346), adults (age ≥16; *n* = 274), ALL, and AML largely duplicated the same results for RRM (Tables S3–S6).

CR1 (HR 1.38; 95% CI 1.16–1.64; *p* < 0.001) and higher CD34^+^ cell dosage (HR 1.16 per 10^5^/kg; 95% CI 1.10 – 1.22; *p* < 0.001) were significant favorable factors that predicted earlier NE (Table S2). The 75th‐percentile time of NE for the most favorable subgroup (CD34^+^ cell dosage ≥0.83 × 10^5^/kg and CR1) was 18 days posttransplant (Figure 2B), 9 days earlier than the least favorable subgroup (CD34^+^ cell dosage < 0.83 × 10^5^/kg and not CR1).

We found that CD34^+^ cell dosage ≥0.83 × 10^5^/kg partially compensated for HLA mismatch ≥4/10 in reducing NRM (HR 0.41; 95% CI, 0.19–0.90; *p* = 0.025; Figure 2C) but not for lack of “KIR‐B/HLA‐C synergy” in reducing RRM (HR 0.71; 95% CI, 0.36–1.37; *p* = 0.30; Figure 2D).

Synthesizing all the above results, in our study cohort, “HLA mismatch ≤3/10, KIR‐B/HLA‐C synergistic” donors (HR 0.05; 95% CI 0.007–0.41; *p* = 0.005) were the most favorable for OS, followed by “HLA mismatch ≤3/10, not KIR‐B/HLA‐C synergistic” (HR 0.27; 95% CI 0.14–0.51; *p* < 0.001), and “HLA mismatch ≥4/10, CD34^+^ cell dosage ≥0.83 × 10^5^/kg” (HR 0.35; 95% CI 0.18–0.69; *p* = 0.003) donors (Figure 2E). The cohort analyzed in this study might be too small for establishing the minimum required CD34^+^ cell dosage in “HLA mismatch ≤3/10, not KIR‐B/HLA‐C synergistic” cases; we, however, would like to point out that the lowest CD34^+^ cell dosage among such cases in the studied cohort was 0.17 × 10^5^/kg (*n* = 1), and this patient (male, age 25.5, intermediate‐risk AML, not CR status at transplant) had not suffered from relapse or mortality at the time of loss of follow‐up (748 days post‐transplant).

## 4 DISCUSSION

Clinical outcome in UCBT especially depends on transplantation center experience [16]. Although our results might not extrapolate to other centers, our data were not susceptible to center‐to‐center variations.

To firmly establish the minimum required CD34^+^ cell dosage, more data will be needed. Nonetheless, our study strongly supports that CD34^+^ cell dosage < 0.83 × 10^5^/kg is permissible for Chinese‐ethnic patients when HLA mismatch is ≤3/10. If this observation can be extrapolated to other ethnicities, we will be one step closer towards eliminating racial disparities in access to CB transplants. Non‐European‐ethnic patients have lower chance of finding CB units with ≥1.5 × 10^5^/kg CD34^+^ cell dosage [17], and African‐heritage patients might have less haploidentical donors and are therefore more dependent on access to CB allografts [18].

To our best knowledge, our study is the first to report that “KIR‐B/HLA‐C synergy” prevents RRM in UCBT. By definition, “KIR‐B/HLA‐C synergy” requires donor‐recipient mismatch at the HLA‐C locus, which is plausible in UCBT due to its relatively high tolerance of HLA mismatch. ≈61% and ≈34% of Chinese are C1/C1 and C1/C2, respectively; therefore, the most probable “KIR‐B/HLA‐C synergistic” UCBT would be “TelB^+^ and C1/C2” donors for C1/C1 recipients. We caution that the utility of “KIR‐B/HLA‐C synergy” has not been prospectively validated in UCBT.

In conclusion, the minimum required CD34^+^ cell dosage can possibly be revised to broaden access to UCBT, especially for adult patients. Moreover, natural killer alloreactivity has the potential to suppress relapse‐related mortality in some patients of acute leukemia, and donor KIR genotyping should be considered during unit selection.

## AUTHOR CONTRIBUTIONS

Z Sun and JC are the co‐senior authors and jointly conceived the study. YJ, HL, KS, LG, WY, XW, BT, XZ, GS, and PQ collected the original diagnostic and clinical data. XL and YH coordinated the study, with assistance from Z Song. Z Sun, YH, JY, XL, XG, and YF extracted the data and performed data curation. Z Sun and JC reviewed the final dataset. YH, XG, YF, WZ, and XL performed the analyses with assistance from SQ and QS. JC wrote the manuscript. YH, YF, XG, XL, and WZ generated the tables and figures. Z Sun and YJ participated in manuscript revision. All of the authors reviewed and approved the manuscript.

## CONFLICT OF INTEREST STATEMENT

The authors declare that there is no conflict of interest that could be perceived as prejudicing the impartiality of the research reported.

## ETHICS STATEMENT

The study was approved by the academic committee (IIT2021042) at the Institute of Hematology, Chinese Academy of Medical Sciences (IHCAMS) and the ethics review committees at both the IHCAMS and the FAHUSTC (QTJC2022026‐EC‐1 and 2022‐RE‐070, respectively). All the adult patients and the guardians of the pediatric patients signed an informed consent form that permitted the patients’ samples or data to be utilized for research.

## Supporting information

Supporting Information

## Data Availability

Our cohort data are available to other researchers upon reasonable request addressed to Z Sun via email. Z Sun is the primary contact of this study after publication.
