# Supplementary material for: Refining eligibility criteria of unit selection for myeloablative cord blood transplantation in acute leukemia: Real‐world experience of a referral center
Source: EJHaem. 2023 Apr 25;4(2):470–5. doi: 10.1002/jha2.703 (PMC10188464; doi:10.1002/jha2.703)
Supplement: Supplementary file 1 — Supporting Information [file JHA2-4-470-s001.pdf]

Supporting Information

SUPPLEMENTARY METHODS

HLA Typing

HLA-A, -B, -C, -DRB1, and -DQB1 of the donors and patients were typed at high resolution (at least "4 digits"). HLA mismatch was calculated at the allele level in the graft-versus-host direction (that is, absence of a recipient allele in the donor).

NK Alloreactivity Assessment

To avoid ambiguity, we detail how we imputed NK alloreactivity as follows.

The inhibitory KIR ligand status was calculated based on HLA-A, -B, and -C[1] using our custom-written R scripts: type "C1" HLA-C was defined as the presence of HLA-C*01/*03/*07/*08/*12/*14/*16; type "C2" HLA-C, the presence of HLA-C*02/*04/*05/*06/*15/*17/*18; type "Bw4" HLA-A or -B[2], the presence of HLA epitope B5, B5102, B5103, B13, B17, B27, B37, B38(16), B44(12), B47, B49(21), B51(5), B52(5), B53, B57(17), B58(17), B59, B63(15), B77(15), A9, A23(9), A24(9), A2403, A25(10), or A32(19); and type HLA-A3/A11, the presence of HLA epitope A3 or A11. When determining the presence/absence of inhibitory KIR ligand types "Bw4" and "A3/A11", we used the correspondence table between serologically defined antigens and HLA alleles described in ref. [3]. Inhibitory KIR ligand mismatch was defined in the graft-versus-host direction. (For instance, C1 ligand graft-versus-host mismatch was defined as when a type C1 ligand was present in the donor but not in the recipient.)

All the donors were genotyped for the presence/absence of KIR genes (KIR2DL1, KIR2DL2, KIR2DL3, KIR2DL4, KIR2DL5, KIR2DS1, KIR3DS1, KIR2DS2, KIR2DS3, KIR2DS4, KIR2DS5, KIR3DL1, KIR3DL2, KIR3DL3, KIR3DP1) using sequence specific oligonucleotide probes after PCR amplification. Based on the linkage disequilibrium structure of the KIR genes in our study cohort, presence of "CenB" was defined as presence of any of these two genes: KIR2DL2 and KIR2DS2; presence of "TelB" was defined as presence of $\geq$2 of these three genes: KIR2DS1, KIR3DS1, and KIR2DL5.

Statistical analysis

Multivariate analysis of overall survival was conducted with Cox regression using the "coxph" function in R. In multivariate analyses of the other types of events (relapse, relapse-related mortality (RRM), non-relapse mortality (NRM), and neutrophil engraftment), we corrected for competing risks. Relapse and NRM were treated as mutual competing risks for each other. NRM was treated as a competing risk for RRM. All-cause death was treated as a competing risk for neutrophil engraftment. Proportional hazards models for subdistributions of competing risks were computed

with the Fine-Gray model using the “crr” function in R, while cumulative incidence curves were compared with the Gray method using the “cuminc” function in R.

References:

1. Heidenreich S, Kroger N. Reduction of Relapse after Unrelated Donor Stem Cell Transplantation by KIR-Based Graft Selection. Front Immunol. 2017;8:41.
2. HLA Antigens: Bw4 and Bw6 associated specificities [Available from: https://hla.alleles.org/antigens/bw46.html.
3. Holdsworth R, Hurley CK, Marsh SG, Lau M, Noreen HJ, Kempenich JH, et al. The HLA dictionary 2008: a summary of HLA-A, -B, -C, -DRB1/3/4/5, and -DQB1 alleles and their association with serologically defined HLA-A, -B, -C, -DR, and -DQ antigens. Tissue Antigens. 2009;73(2):95-170.

Table S1. Characteristics of the study cohort.

	UCBT (n = 620)	MSD HSCT (n = 75)	P
**Year at transplant, n (%)**			<0.001 †
2015	25 (4.0)	10 (13)	
2016	51 (8.2)	5 (7)	
2017	110 (17.7)	20 (27)	
2018	132 (21.3)	19 (25)	
2019	163 (26.3)	10 (13)	
2020	139 (22.4)	11 (15)	
**Age at transplant, years, median (range)**	13.7 (0.7 – 62.3)	35.4 (4.5 – 56.8)	<0.001 *
**Biological sex, n (%)**			0.52 †
Male	343 (55.3)	38 (51)	
Female	277 (44.7)	37 (49)	
**Primary disease, n (%)**			0.03 †
Low-risk ALL	123 (19.8)	10 (13)	
High-risk ALL	147 (23.7)	17 (23)	
Standard-risk AML	77 (12.4)	9 (12)	
Intermediate-risk AML	175 (28.2)	34 (45)	
High-risk AML	88 (14.2)	4 (5)	
MPAL	10 (1.6)	1 (1)	
**Disease status at transplant, n (%)**			0.09 †
CR1	353 (56.9)	51 (68)	
Not CR1	267 (43.1)	24 (32)	
**Conditioning regimen**			NA
MAC	620 (100.0)	75 (100)	
RIC	0 (0.0)	0 (0)	
**HLA mismatch in graft-versus-host direction, n (%)**			<0.001 *
None	31 (5.0)	75 (100)	
1 locus	72 (11.6)	0 (0)	
2 loci	170 (27.4)	0 (0)	
3 loci	189 (30.5)	0 (0)	
4 loci	136 (21.9)	0 (0)	
5 loci	22 (3.5)	0 (0)	
**Presence of donor-specific antibody in the recipient, n (%)**			<0.001 †
Yes	3 (0.5)	0 (0)	
No	601 (96.9)	35 (47)	
Unknown	16 (2.6)	40 (53)	
**ABO match, n (%)**			0.02 †
Matched	204 (32.9)	38 (51)	
Major mismatch	165 (26.6)	13 (17)	
Minor mismatch	171 (27.6)	14 (19)	

Bidirectional mismatch	80 (12.9)	10 (13)	
**Inhibitory KIR ligand mismatch in graft-versus-host direction, n (%)**			
"C1" HLA-C	21 (3.4)	0 (0)	NA
"C2" HLA-C	39 (6.3)	0 (0)	NA
"Bw4" HLA-A or –B	23 (3.7)	0 (0)	NA
HLA-A3 or -A11	48 (7.7)	0 (0)	NA
Any of the above	119 (19.2)	0 (0)	NA
**Presence of donor KIR CenB**			NA
Yes	144 (23.2)	0 (0)	
No	476 (76.8)	0 (0)	
Unknown	0 (0.0)	75 (100)	
**Presence of donor KIR TelB**			NA
Yes	263 (42.4)	0 (0)	
No	357 (57.6)	0 (0)	
Unknown	0 (0.0)	75 (100)	
**Infused $CD34^+$ cell dosage, 10^5/kg, median (range)**	1.90 (0.17 – 10.90)	50.9 (12.6 – 128.7)	<0.001 *
**aGVHD prophylaxis, n (%)**			<0.001 †
CsA + MMF	613 (98.9)	61 (81)	
CsA + MMF + CTX	0 (0.0)	1 (1)	
CsA + MMF + MTX	7 (1.1)	13 (17)	
**Neutrophil engraftment by day 14 post-transplant, n (%)**	187 (30.2)	71 (95)	<0.001 †
**Follow-up duration since transplant, days, median (range)**	931 (2 – 2636)	1151 (19 – 2620)	0.14 *
**Severe aGVHD within 100 days, % (based on competing risk analysis)**	16	5	0.011 ‡
**Overall survival, % (based on the Kaplan-Meier method)**			0.43 §
6 months	86	85	
1 year	79	80	
2 years	75	72	
**Relapse, % (based on competing risk analysis)**			0.16 ‡
6 months	8	13	
1 year	14	19	
2 years	18	26	
**Relapse-related mortality, % (based on competing risk analysis)**			0.012 ‡
6 months	3	8	
1 year	7	11	
2 years	11	17	
**Non-relapse mortality, % (based on competing risk analysis)**			0.28 ‡
6 months	11	7	
1 year	14	9	
2 years	14	11	

Abbreviations: aGVHD, acute graft-versus-host disease; AML, acute myeloid leukemia; ALL, acute lymphoblastic leukemia; CenB, centromeric haplotype-B; CR, complete remission; CsA, cyclosporine A; CTX, cyclophosphamide; HLA, human leukocyte antigen; KIR, killer-cell immunoglobulin-like receptor; MAC, myeloablative conditioning; MMF, mycophenolate mofetil; MPAL, mixed phenotype acute leukemia; MTX, methotrexate; NA, not applicable; RIC, reduced-intensity conditioning; TelB, telomeric haplotype-B.

[*] t test.
[†] Chi-squared test.
[‡] Gray test.
[§] Log-rank test.

Table S2. Multivariate analyses for the determining factors of relapse, relapse-related mortality, non-relapse mortality, overall survival, and neutrophil engraftment (n = 620).

	**Relapse**		**Relapse-related mortality**		**Non-relapse mortality**		**Overall survival**		**Neutrophil engraftment**	
	HR (95% C.I.)	P	HR (95% C.I.)	P	HR (95% C.I.)	P	HR (95% C.I.)	P	HR (95% C.I.)	P
**Year at transplant (per year)**	0.93 (0.81 – 1.06)	0.26	0.87 (0.73 – 1.03)	0.10	0.90 (0.79 – 1.04)	0.14	0.89 (0.80 – 0.99)	0.03	1.05 (0.98 – 1.12)	0.18
**Biological sex**										
Female vs. male	0.84 (0.58 – 1.23)	0.38	0.79 (0.49 – 1.27)	0.33	1.10 (0.71 – 1.71)	0.67	0.95 (0.70 – 1.30)	0.77	1.01 (0.86 – 1.19)	0.90
**Age at transplant (per year)**	1.00 (0.99 – 1.02)	0.71	1.00 (0.98 – 1.02)	0.92	1.02 (1.00 – 1.04)	0.02	1.01 (1.00 – 1.02)	0.03	1.00 (0.99 – 1.00)	0.19
**Primary disease**										
Low-risk ALL	1.00		1.00		1.00		1.00		1.00	
High-risk ALL	1.80 (1.06 – 3.06)	0.03	2.00 (1.00 – 3.99)	0.048	0.37 (0.19 – 0.71)	0.003	0.80 (0.51 – 1.26)	0.33	0.85 (0.65 – 1.09)	0.20
Standard-risk AML	0.55 (0.24 – 1.23)	0.14	0.52 (0.17 – 1.56)	0.25	0.60 (0.30 – 1.22)	0.16	0.57 (0.31 – 1.03)	0.06	0.91 (0.67 – 1.22)	0.52
Intermediate-risk AML	1.14 (0.65 – 2.02)	0.65	1.69 (0.86 – 3.33)	0.13	0.43 (0.23 – 0.81)	0.010	0.75 (0.48 – 1.18)	0.21	0.73 (0.57 – 0.95)	0.02
High-risk AML	0.78 (0.38 – 1.60)	0.50	1.05 (0.44 – 2.50)	0.91	0.63 (0.31 – 1.28)	0.20	0.79 (0.46 – 1.34)	0.38	0.75 (0.56 – 1.01)	0.06
MPAL	3.62 (1.47 – 8.91)	0.005	3.31 (0.66 – 16.60)	0.14	2.17 (0.68 – 6.92)	0.19	3.22 (1.30 – 7.94)	0.011	0.87 (0.45 – 1.70)	0.69
**Disease status at transplant**										
CR1 vs. not CR1	0.35 (0.24 – 0.52)	<0.001	0.33 (0.20 – 0.53)	<0.001	0.70 (0.44 – 1.10)	0.12	0.49 (0.35 – 0.67)	<0.001	1.38 (1.16 – 1.64)	<0.001
**Conditioning regimen**										
MAC vs. RIC*	—		—		—		—		—	
**Infused CD34$^+$ cell dosage (per 10^5 cells/kg)**	0.99 (0.87 – 1.13)	0.91	1.04 (0.90 – 1.20)	0.62	0.98 (0.82 – 1.17)	0.80	1.00 (0.90 – 1.12)	0.94	1.16 (1.10 – 1.22)	<0.001

**ABO match**										
Major mismatch vs. bidirectional mismatch	1.22 (0.66 – 2.25)	0.52	1.42 (0.63 – 3.21)	0.39	1.32 (0.59 – 2.92)	0.50	1.48 (0.85 – 2.58)	0.17	1.04 (0.79 – 1.37)	0.78
Minor mismatch vs. bidirectional mismatch	1.06 (0.57 – 1.96)	0.86	1.29 (0.57 – 2.93)	0.55	1.22 (0.54 – 2.74)	0.64	1.34 (0.76 – 2.35)	0.31	1.18 (0.90 – 1.55)	0.24
Match vs. bidirectional mismatch	0.94 (0.50 – 1.76)	0.84	1.07 (0.47 – 2.48)	0.87	1.63 (0.75 – 3.53)	0.22	1.45 (0.84 – 2.53)	0.18	0.87 (0.66 – 1.14)	0.32
**HLA mismatch in graft-versus-host direction**										
≤3/10 vs. ≥4/10	1.35 (0.88 – 2.09)	0.17	1.35 (0.78 – 2.37)	0.29	0.46 (0.30 – 0.72)	<0.001	0.72 (0.51 – 1.00)	0.052	1.27 (1.05 – 1.54)	0.015
**Inhibitory KIR ligand mismatch in graft-versus-host direction**	0.84 (0.52 – 1.37)	0.49	0.80 (0.41 – 1.56)	0.51	1.02 (0.55 – 1.90)	0.95	0.92 (0.58 – 1.45)	0.71	1.07 (0.85 – 1.35)	0.57
**Presence of donor CenB**	0.69 (0.42 – 1.13)	0.14	0.66 (0.37 – 1.19)	0.17	1.18 (0.69 – 2.00)	0.55	0.88 (0.59 – 1.30)	0.52	0.99 (0.80 – 1.23)	0.94
**Presence of donor TelB**	0.98 (0.68 – 1.43)	0.93	1.62 (1.03 – 2.54)	0.04	1.30 (0.83 – 2.03)	0.25	1.51 (1.09 – 2.09)	0.012	1.16 (0.97 – 1.39)	0.11
**Donor CenB and type C1 ligand graft-versus-host mismatch**	1.72 (0.10 – 28.71)	0.71	<0.01 (<0.01)	<0.001	1.41 (0.17 – 11.43)	0.75	1.06 (0.13 – 8.49)	0.95	2.24 (0.82 – 6.11)	0.11
**Donor CenB and type C2 ligand graft-versus-host mismatch**	1.40 (0.14 – 14.18)	0.77	3.73 (0.42 – 33.20)	0.24	1.35 (0.30 – 6.00)	0.70	2.30 (0.69 – 7.61)	0.17	0.59 (0.29 – 1.22)	0.16
**Donor TelB and type C1 ligand graft-versus-host mismatch**	0.79 (0.06 – 9.69)	0.86	<0.01 (<0.01)	<0.001	0.98 (0.15 – 6.30)	0.98	0.50 (0.06 – 3.88)	0.51	1.08 (0.48 – 2.41)	0.85
**Donor TelB and type C2 ligand graft-versus-host mismatch**	0.32 (0.04 – 2.88)	0.31	<0.01 (<0.01)	<0.001	0.91 (0.22 – 3.75)	0.90	0.42 (0.12 – 1.53)	0.19	0.89 (0.49 – 1.59)	0.69

Multivariate analysis of overall survival was conducted with Cox regression. In multivariate analyses of the other types of events (relapse, relapse-related mortality, non-relapse mortality, and neutrophil engraftment), we corrected for competing risks using the Fine-Gray model. Hazard ratios less than 0.01 are uniformly shown as “<0.01”, and 95% C.I.'s whose upper limits are <0.01 are shown as “<0.01”. P values less than 0.001 are shown as “<0.001”. All the reported P values were not adjusted for multiple-hypothesis testing.

[*] All the cases in the study cohort were MAC.

Table S3. Pediatric patients (age <16): multivariate analysis for the determining factors of relapse, relapse-related mortality, non-relapse mortality, overall survival, and neutrophil engraftment (n = 346).

	Relapse		Relapse-related mortality		Non-relapse mortality		Overall survival		Neutrophil engraftment	
	HR (95% C.I.)	P	HR (95% C.I.)	P	HR (95% C.I.)	P	HR (95% C.I.)	P	HR (95% C.I.)	P
**Year at transplant (per year)**	0.98 (0.82 – 1.16)	0.79	0.90 (0.72 – 1.13)	0.37	0.99 (0.81 – 1.20)	0.90	0.94 (0.81 – 1.09)	0.44	0.99 (0.90 – 1.08)	0.76
**Biological sex**										
Female vs. male	0.92 (0.53 – 1.61)	0.77	0.98 (0.51 – 1.90)	0.96	1.02 (0.52 – 2.01)	0.96	1.04 (0.66 – 1.62)	0.87	1.00 (0.80 – 1.26)	0.98
**Age at transplant (per year)**	0.99 (0.92 – 1.07)	0.82	0.97 (0.88 – 1.07)	0.55	0.98 (0.90 – 1.07)	0.68	0.97 (0.91 – 1.03)	0.33	1.00 (0.97 – 1.03)	0.84
**Primary disease**										
Low-risk ALL	1.00		1.00		1.00		1.00		1.00	
High-risk ALL	2.27 (1.17 – 4.41)	0.02	2.27 (0.99 – 5.20)	0.052	0.24 (0.08 – 0.71)	0.010	0.79 (0.43 – 1.45)	0.45	0.90 (0.66 – 1.24)	0.53
Standard-risk AML	0.41 (0.09 – 1.78)	0.23	0.69 (0.15 – 3.13)	0.63	0.19 (0.05 – 0.73)	0.02	0.31 (0.11 – 0.90)	0.03	0.93 (0.62 – 1.38)	0.71
Intermediate-risk AML	1.35 (0.64 – 2.86)	0.43	1.93 (0.84 – 4.47)	0.12	0.39 (0.15 – 1.04)	0.06	0.83 (0.44 – 1.55)	0.55	0.82 (0.58 – 1.14)	0.23
High-risk AML	1.13 (0.44 – 2.91)	0.80	1.34 (0.44 – 4.05)	0.61	0.61 (0.22 – 1.75)	0.36	0.93 (0.45 – 1.93)	0.85	0.80 (0.55 – 1.16)	0.24
MPAL	2.11	0.18	<0.01	<0.001	5.80	0.005	3.45	0.12	1.20	0.77

	(0.72 – 6.18)		(<0.01)		(1.71 – 19.70)		(0.73 – 16.15)		(0.37 – 3.87)	
**Disease status at transplant**										
CR1 vs. not CR1	0.28 (0.16 – 0.49)	<0.001	0.32 (0.17 – 0.62)	<0.001	1.28 (0.57 – 2.84)	0.55	0.64 (0.40 – 1.04)	0.07	1.26 (1.00 – 1.60)	0.054
**Conditioning regimen**										
MAC vs. RIC*	—		—		—		—		—	
**Infused CD34$^+$ cell dosage (per 10^5 cells/kg)**	0.99 (0.85 – 1.14)	0.85	1.01 (0.86 – 1.18)	0.95	1.04 (0.89 – 1.23)	0.61	1.01 (0.90 – 1.15)	0.83	1.14 (1.07 – 1.21)	<0.001
**ABO match**										
Major mismatch vs. bidirectional mismatch	1.15 (0.50 – 2.67)	0.74	1.31 (0.48 – 3.57)	0.60	1.30 (0.36 – 4.69)	0.69	1.58 (0.71 – 3.50)	0.27	1.02 (0.71 – 1.48)	0.91
Minor mismatch vs. bidirectional mismatch	0.79 (0.33 – 1.89)	0.60	0.62 (0.19 – 1.96)	0.41	1.56 (0.44 – 5.60)	0.50	1.13 (0.48 – 2.67)	0.78	1.17 (0.81 – 1.69)	0.41
Match vs. bidirectional mismatch	0.89 (0.38 – 2.10)	0.79	1.02 (0.36 – 2.92)	0.97	1.91 (0.56 – 6.49)	0.30	1.72 (0.79 – 3.78)	0.17	0.95 (0.66 – 1.36)	0.77
**HLA mismatch in graft-versus-host direction**										
≤3/10 vs. ≥4/10	1.19 (0.60 – 2.37)	0.62	1.34 (0.53 – 3.36)	0.54	0.73 (0.37 – 1.47)	0.38	1.00 (0.59 – 1.69)	0.99	1.32 (1.01 – 1.74)	0.04
**Inhibitory KIR ligand mismatch in graft-versus-host direction**	0.66 (0.32 – 1.34)	0.25	0.71 (0.28 – 1.82)	0.48	2.06 (1.04 – 4.07)	0.04	1.29 (0.72 – 2.32)	0.39	1.03 (0.76 – 1.41)	0.84
**Presence of donor CenB**	0.63 (0.30 – 1.30)	0.21	0.57 (0.26 – 1.28)	0.17	1.35 (0.64 – 2.88)	0.43	0.88 (0.50 – 1.55)	0.66	0.93 (0.70 – 1.24)	0.63
**Presence of donor TelB**	1.33 (0.77 – 2.30)	0.30	2.14 (1.16 – 3.96)	0.02	1.19 (0.60 – 2.37)	0.61	1.63 (1.01 – 2.63)	0.04	1.37 (1.07 – 1.75)	0.013

**Donor CenB and type C1 ligand graft-versus-host mismatch**	<0.01 (<0.01)	<0.001	<0.01 (<0.01)	<0.001	<0.01 (<0.01)	<0.001	<0.01 (<0.01 – >10.00)	>0.99	9.97 (1.28 – 77.62)	0.03
**Donor CenB and type C2 ligand graft-versus-host mismatch**	<0.01 (<0.01)	<0.001	<0.01 (<0.01)	<0.001	2.02 (0.09 – 44.90)	0.66	1.38 (0.14 – 13.94)	0.79	0.73 (0.21 – 2.52)	0.62
**Donor TelB and type C1 ligand graft-versus-host mismatch**	3.81 (0.51 – 28.40)	0.19	<0.01 (<0.01)	<0.001	<0.01 (<0.01)	<0.001	<0.01 (<0.01 – >10.00)	>0.99	1.88 (0.43 – 8.16)	0.40
**Donor TelB and type C2 ligand graft-versus-host mismatch**	<0.01 (<0.01)	<0.001	<0.01 (<0.01)	<0.001	0.35 (0.02 – 8.23)	0.52	0.23 (0.02 – 2.16)	0.20	1.47 (0.56 – 3.85)	0.43

Multivariate analysis of overall survival was conducted with Cox regression. In multivariate analyses of the other types of events (relapse, relapse-related mortality, non-relapse mortality, and neutrophil engraftment), we corrected for competing risks using the Fine-Gray model. Hazard ratios less than 0.01 are uniformly shown as "<0.01", and 95% C.I.'s whose upper limits are <0.01 are shown as "<0.01". P values less than 0.001 are shown as "<0.001". All the reported P values were not adjusted for multiple-hypothesis testing.

[*] All the cases in the study cohort were MAC.

Table S4. Adult patients (age ≥16): multivariate analysis for the determining factors of relapse, relapse-related mortality, non-relapse mortality, overall survival, and neutrophil engraftment (n = 274).

	**Relapse**		**Relapse-related mortality**		**Non-relapse mortality**		**Overall survival**		**Neutrophil engraftment**	
	HR (95% C.I.)	P	HR (95% C.I.)	P	HR (95% C.I.)	P	HR (95% C.I.)	P	HR (95% C.I.)	P
**Year at transplant (per year)**	0.85 (0.68 – 1.07)	0.16	— †		— †		0.78 (0.66 – 0.92)	0.004	1.15 (1.04 – 1.27)	0.006
**Biological sex**										
Female vs. male	0.70 (0.38 – 1.27)	0.24	0.60 (0.28 – 1.29)	0.19	0.96 (0.50 – 1.85)	0.91	0.72 (0.46 – 1.14)	0.16	1.11 (0.85 – 1.45)	0.44
**Age at transplant (per year)**	0.99 (0.96 – 1.01)	0.30	1.00 (0.97 – 1.04)	0.79	1.02 (0.99 – 1.05)	0.13	1.02 (1.00 – 1.04)	0.10	0.99 (0.98 – 1.00)	0.21
**Primary disease**										
Low-risk ALL	1.00		1.00		1.00		1.00		1.00	
High-risk ALL	1.40 (0.55 – 3.55)	0.48	1.59 (0.40 – 6.37)	0.51	0.42 (0.16 – 1.09)	0.07	0.84 (0.38 – 1.83)	0.65	0.74 (0.46 – 1.19)	0.22
Standard-risk AML	0.60 (0.20 – 1.76)	0.35	0.37 (0.06 – 2.17)	0.27	0.77 (0.28 – 2.16)	0.62	0.74 (0.31 – 1.74)	0.49	0.72 (0.43 – 1.21)	0.22
Intermediate-risk AML	1.13 (0.49 – 2.61)	0.78	1.26 (0.38 – 4.23)	0.71	0.36 (0.14 – 0.92)	0.03	0.66 (0.30 – 1.44)	0.30	0.61 (0.39 – 0.98)	0.04
High-risk AML	0.48 (0.14 – 1.63)	0.24	0.60 (0.13 – 2.78)	0.51	0.49 (0.18 – 1.34)	0.16	0.57 (0.24 – 1.37)	0.21	0.75 (0.45 – 1.25)	0.27
MPAL	4.00	0.04	4.44	0.15	1.33	0.76	2.98	0.09	0.63	0.30

	(1.06 – 15.20)		(0.58 – 34.20)		(0.22 – 8.24)		(0.84 – 10.51)		(0.26 – 1.50)	
**Disease status at transplant**										
CR1 vs. not CR1	0.34 (0.18 – 0.63)	<0.001	0.25 (0.10 – 0.59)	0.002	0.48 (0.25 – 0.94)	0.03	0.32 (0.19 – 0.52)	<0.001	1.60 (1.22 – 2.10)	<0.001
**Conditioning regimen**										
MAC vs. RIC*	—		—		—		—		—	
**Infused CD34+ cell dosage (per 10^5 cells/kg)**	1.14 (0.86 – 1.50)	0.36	1.15 (0.78 – 1.70)	0.49	0.76 (0.53 – 1.10)	0.15	0.87 (0.67 – 1.13)	0.30	1.29 (1.14 – 1.45)	<0.001
**ABO match**										
Major mismatch vs. bidirectional mismatch	1.21 (0.48 – 3.04)	0.69	1.22 (0.31 – 4.74)	0.78	1.51 (0.46 – 4.92)	0.49	1.49 (0.65 – 3.45)	0.35	0.92 (0.60 – 1.42)	0.70
Minor mismatch vs. bidirectional mismatch	1.13 (0.44 – 2.86)	0.80	2.08 (0.57 – 7.66)	0.27	1.24 (0.39 – 3.93)	0.72	1.73 (0.76 – 3.95)	0.19	1.14 (0.75 – 1.74)	0.54
Match vs. bidirectional mismatch	0.87 (0.35 – 2.17)	0.76	0.78 (0.20 – 3.05)	0.72	1.41 (0.46 – 4.25)	0.55	1.20 (0.52 – 2.79)	0.67	0.73 (0.47 – 1.12)	0.15
**HLA mismatch in graft-versus-host direction**										
≤3/10 vs. ≥4/10	1.40 (0.76 – 2.57)	0.28	1.08 (0.51 – 2.29)	0.84	0.28 (0.15 – 0.52)	<0.001	0.52 (0.33 – 0.82)	0.005	1.34 (1.00 – 1.80)	0.048
**Inhibitory KIR ligand mismatch in graft-versus-host direction**	1.19 (0.57 – 2.53)	0.64	0.99 (0.39 – 2.52)	0.98	0.48 (0.12 – 1.82)	0.28	0.60 (0.27 – 1.34)	0.21	1.11 (0.76 – 1.64)	0.59
**Presence of donor CenB**	0.64 (0.30 – 1.38)	0.26	0.69 (0.26 – 1.81)	0.45	1.21 (0.53 – 2.77)	0.65	0.96 (0.53 – 1.74)	0.88	1.09 (0.77 – 1.56)	0.62
**Presence of donor TelB**	0.76 (0.43 – 1.34)	0.34	1.30 (0.59 – 2.87)	0.51	1.45 (0.72 – 2.89)	0.30	1.55 (0.96 – 2.51)	0.08	0.91 (0.69 – 1.20)	0.50
**Donor CenB and type C1 ligand graft-versus-host mismatch**	6.49 (0.33 – 127.00)	0.22	<0.01 (<0.01)	<0.001	5.11 (0.55 – 47.52)	0.15	2.89 (0.27 – 31.01)	0.38	1.61 (0.50 – 5.20)	0.42

**Donor CenB and type C2 ligand graft-versus-host mismatch**	2.70 (0.28 – 25.80)	0.39	3.95 (0.34 – 46.20)	0.27	3.42 (0.45 – 26.33)	0.24	3.56 (0.71 – 17.85)	0.12	0.29 (0.10 – 0.83)	0.02
**Donor TelB and type C1 ligand graft-versus-host mismatch**	<0.01 (<0.01)	<0.001	<0.01 (<0.01)	<0.001	1.95 (0.28 – 13.49)	0.50	1.05 (0.10 – 10.82)	0.97	1.14 (0.43 – 3.00)	0.79
**Donor TelB and type C2 ligand graft-versus-host mismatch**	0.60 (0.07 – 5.15)	0.64	<0.01 (<0.01)	<0.001	2.47 (0.43 – 14.32)	0.31	1.85 (0.36 – 9.57)	0.46	0.50 (0.21 – 1.21)	0.13

Multivariate analysis of overall survival was conducted with Cox regression. In multivariate analyses of the other types of events (relapse, relapse-related mortality, non-relapse mortality, and neutrophil engraftment), we corrected for competing risks using the Fine-Gray model. Hazard ratios less than 0.01 are uniformly shown as “<0.01”, and 95% C.I.'s whose upper limits are <0.01 are shown as “<0.01”. P values less than 0.001 are shown as “<0.001”. All the reported P values were not adjusted for multiple-hypothesis testing.

[*] All the cases in the study cohort were MAC.

[†] Incomputable due to numerical singularity.

Table S5. Acute lymphoblastic leukemia: multivariate analysis for the determining factors of relapse, relapse-related mortality, non-relapse mortality, overall survival, and neutrophil engraftment (n = 270).

	**Relapse**		**Relapse-related mortality**		**Non-relapse mortality**		**Overall survival**		**Neutrophil engraftment**	
	HR (95% C.I.)	P	HR (95% C.I.)	P	HR (95% C.I.)	P	HR (95% C.I.)	P	HR (95% C.I.)	P
**Year at transplant (per year)**	— †		— †		0.83 (0.69 – 1.02)	0.07	0.83 (0.72 – 0.97)	0.02	1.01 (0.92 – 1.11)	0.80
**Biological sex**										
Female vs. male	0.81 (0.46 – 1.42)	0.46	0.80 (0.39 – 1.63)	0.54	1.31 (0.69 – 2.51)	0.41	1.15 (0.73 – 1.81)	0.54	0.91 (0.69 – 1.19)	0.47
**Age at transplant (per year)**	1.00 (0.97 – 1.02)	0.73	0.99 (0.97 – 1.02)	0.60	1.03 (1.00 – 1.06)	0.04	1.01 (0.99 – 1.03)	0.20	1.00 (0.99 – 1.01)	0.61
**Primary disease**										
Low-risk ALL	1.00		1.00		1.00		1.00		1.00	
High-risk ALL	2.11 (1.17 – 3.81)	0.013	2.28 (1.06 – 4.90)	0.03	0.25 (0.12 – 0.52)	<0.001	0.75 (0.46 – 1.22)	0.24	0.88 (0.66 – 1.16)	0.36
**Disease status at transplant**										
CR1 vs. not CR1	0.21 (0.11 – 0.39)	<0.001	0.17 (0.07 – 0.40)	<0.001	1.36 (0.71 – 2.63)	0.36	0.55 (0.34 – 0.89)	0.014	1.26 (0.96 – 1.65)	0.10
**Conditioning regimen**										
MAC vs. RIC*	—		—		—		—		—	
**Infused CD34$^+$ cell dosage (per 10^5 cells/kg)**	0.88 (0.71 – 1.10)	0.27	0.84 (0.63 – 1.13)	0.25	0.77 (0.60 – 1.00)	0.045	0.77 (0.63 – 0.95)	0.014	1.26 (1.15 – 1.39)	<0.001

**ABO match**										
Major mismatch vs. bidirectional mismatch	1.02 (0.41 – 2.51)	0.97	1.23 (0.44 – 3.47)	0.69	1.00 (0.29 – 3.44)	>0.99	1.19 (0.52 – 2.68)	0.68	1.17 (0.76 – 1.80)	0.47
Minor mismatch vs. bidirectional mismatch	0.80 (0.32 – 2.02)	0.64	0.73 (0.23 – 2.32)	0.59	1.22 (0.34 – 4.29)	0.76	0.89 (0.38 – 2.09)	0.79	1.24 (0.81 – 1.91)	0.33
Match vs. bidirectional mismatch	0.63 (0.25 – 1.56)	0.31	0.71 (0.24 – 2.11)	0.54	1.24 (0.39 – 3.89)	0.72	1.02 (0.46 – 2.29)	0.96	1.01 (0.66 – 1.52)	0.98
**HLA mismatch in graft-versus-host direction**										
≤3/10 vs. ≥4/10	1.26 (0.69 – 2.30)	0.44	1.34 (0.57 – 3.17)	0.50	0.43 (0.23 – 0.82)	0.011	0.68 (0.42 – 1.09)	0.11	1.31 (0.98 – 1.76)	0.07
**Inhibitory KIR ligand mismatch in graft-versus-host direction**	0.78 (0.37 – 1.61)	0.49	0.71 (0.23 – 2.18)	0.55	1.79 (0.78 – 4.10)	0.17	1.18 (0.62 – 2.25)	0.60	0.86 (0.60 – 1.23)	0.40
**Presence of donor CenB**	0.77 (0.39 – 1.53)	0.46	0.75 (0.35 – 1.59)	0.45	1.38 (0.69 – 2.77)	0.36	1.00 (0.59 – 1.70)	>0.99	0.97 (0.71 – 1.34)	0.86
**Presence of donor TelB**	0.98 (0.55 – 1.75)	0.95	1.73 (0.87 – 3.44)	0.12	1.79 (0.98 – 3.25)	0.056	1.87 (1.17 – 2.98)	0.0092	1.21 (0.92 – 1.59)	0.18
**Donor CenB and type C1 ligand graft-versus-host mismatch**	143.41 (34.49 – 596.25)	<0.001	<0.01 (<0.01)	<0.001	<0.01 (<0.01)	<0.001	<0.01 (<0.01 – >10.00)	>0.99	4.06 (0.51 – 32.06)	0.18
**Donor CenB and type C2 ligand graft-versus-host mismatch**	3.70 (0.18 – 75.02)	0.39	7.83 (0.29 – 210.00)	0.22	<0.01 (<0.01)	<0.001	1.06 (0.12 – 9.31)	0.96	1.15 (0.25 – 5.31)	0.86
**Donor TelB and type C1 ligand graft-versus-host mismatch**	5.24 (0.86 – 31.95)	0.07	<0.01 (<0.01)	<0.001	<0.01 (<0.01)	<0.001	<0.01 (<0.01 – >10.00)	>0.99	2.60 (0.59 – 11.39)	0.21
**Donor TelB and type C2 ligand graft-versus-host mismatch**	0.73 (0.08 – 6.78)	0.78	<0.01 (<0.01)	<0.001	0.23 (0.03 – 2.04)	0.19	0.22 (0.03 – 1.79)	0.16	0.82 (0.36 – 1.85)	0.63

Multivariate analysis of overall survival was conducted with Cox regression. In multivariate analyses of the other types of events (relapse, relapse-related mortality, non-relapse mortality, and neutrophil engraftment), we corrected for competing risks using the Fine-Gray model. Hazard ratios less than 0.01 are uniformly shown as “<0.01”, and 95% C.I.'s whose upper limits are <0.01 are shown as “<0.01”. P values less than 0.001 are shown as “<0.001”. All the reported P values were not adjusted for multiple-hypothesis testing.

[*] All the cases in the study cohort were MAC.

[†] Incomputable due to numerical singularity.

Table S6. Acute myeloid leukemia: multivariate analysis for the determining factors of relapse, relapse-related mortality, non-relapse mortality, overall survival, and neutrophil engraftment (n = 340).

	**Relapse**		**Relapse-related mortality**		**Non-relapse mortality**		**Overall survival**		**Neutrophil engraftment**	
	HR (95% C.I.)	P	HR (95% C.I.)	P	HR (95% C.I.)	P	HR (95% C.I.)	P	HR (95% C.I.)	P
**Year at transplant (per year)**	0.95 (0.75 – 1.20)	0.65	0.84 (0.63 – 1.14)	0.26	1.03 (0.82 – 1.29)	0.82	0.96 (0.80 – 1.14)	0.62	1.10 (1.00 – 1.21)	0.048
**Biological sex**										
Female vs. male	0.95 (0.53 – 1.71)	0.86	0.85 (0.42 – 1.73)	0.65	0.87 (0.45 – 1.66)	0.66	0.87 (0.55 – 1.36)	0.53	1.11 (0.89 – 1.39)	0.35
**Age at transplant (per year)**	1.01 (0.99 – 1.03)	0.27	1.01 (0.99 – 1.04)	0.33	1.02 (1.00 – 1.04)	0.04	1.02 (1.00 – 1.03)	0.02	1.00 (0.99 – 1.00)	0.25
**Primary disease**										
Standard-risk AML	1.00		1.00		1.00		1.00		1.00	
Intermediate-risk AML	1.95 (0.88 – 4.31)	0.10	2.87 (0.94 – 8.74)	0.06	0.81 (0.36 – 1.82)	0.61	1.31 (0.71 – 2.42)	0.38	0.89 (0.67 – 1.19)	0.44
High-risk AML	1.39 (0.56 – 3.45)	0.48	2.06 (0.61 – 6.97)	0.25	1.17 (0.47 – 2.90)	0.74	1.38 (0.70 – 2.73)	0.36	0.91 (0.66 – 1.26)	0.57
**Disease status at transplant**										
CR1 vs. not CR1	0.53 (0.30 – 0.95)	0.03	0.52 (0.25 – 1.06)	0.07	0.36 (0.19 – 0.69)	0.002	0.40 (0.25 – 0.63)	<0.001	1.47 (1.16 – 1.86)	0.0013
**Conditioning regimen**										
MAC vs. RIC*	—		—		—		—		—	
**Infused $CD34^+$ cell dosage (per 10^5 cells/kg)**	1.11 (0.96 – 1.29)	0.16	1.19 (1.01 – 1.14)	0.04	1.06 (0.85 – 1.33)	0.62	1.13 (0.99 – 1.28)	0.06	1.11 (1.04 – 1.19)	0.002
**ABO match**										
Major mismatch vs. bidirectional mismatch	1.23 (0.48 – 3.18)	0.67	1.09 (0.35 – 3.45)	0.88	1.38 (0.44 – 4.29)	0.58	1.28 (0.58 – 2.81)	0.54	0.95 (0.66 – 1.37)	0.79

Minor mismatch vs. bidirectional mismatch	1.15 (0.44 – 2.98)	0.77	1.49 (0.48 – 4.64)	0.49	1.26 (0.43 – 3.70)	0.68	1.37 (0.63 – 2.99)	0.43	1.23 (0.85 – 1.78)	0.27
Match vs. bidirectional mismatch	1.13 (0.44 – 2.92)	0.80	1.12 (0.35 – 3.64)	0.85	1.45 (0.48 – 4.40)	0.51	1.33 (0.61 – 2.89)	0.48	0.78 (0.53 – 1.13)	0.18
**HLA mismatch in graft-versus-host direction**										
≤3/10 vs. ≥4/10	1.28 (0.64 – 2.55)	0.49	1.26 (0.54 – 2.94)	0.60	0.54 (0.28 – 1.05)	0.07	0.74 (0.46 – 1.22)	0.24	1.16 (0.89 – 1.50)	0.29
**Inhibitory KIR ligand mismatch in graft-versus-host direction**	0.91 (0.43 – 1.93)	0.80	1.00 (0.42 – 2.35)	>0.99	0.68 (0.25 – 1.85)	0.45	0.74 (0.37 – 1.48)	0.40	1.29 (0.94 – 1.77)	0.12
**Presence of donor CenB**	0.65 (0.30 – 1.39)	0.26	0.62 (0.26 – 1.44)	0.26	1.01 (0.38 – 2.69)	0.99	0.77 (0.40 – 1.46)	0.42	0.99 (0.73 – 1.35)	0.95
**Presence of donor TelB**	1.06 (0.62 – 1.83)	0.83	1.73 (0.90 – 3.35)	0.10	0.78 (0.36 – 1.69)	0.53	1.16 (0.71 – 1.88)	0.56	1.12 (0.87 – 1.44)	0.37
**Donor CenB and type C1 ligand graft-versus-host mismatch**	<0.01 (<0.01)	<0.001	<0.01 (<0.01)	<0.001	2.16 (0.21 – 22.46)	0.52	1.35 (0.14 – 12.76)	0.79	2.03 (0.64 – 6.49)	0.23
**Donor CenB and type C2 ligand graft-versus-host mismatch**	<0.01 (<0.01)	<0.001	<0.01 (<0.01)	<0.001	4.08 (0.48 – 34.85)	0.20	4.31 (0.77 – 24.16)	0.10	0.19 (0.06 – 0.61)	0.006
**Donor TelB and type C1 ligand graft-versus-host mismatch**	<0.01 (<0.01)	<0.001	<0.01 (<0.01)	<0.001	2.43 (0.38 – 15.56)	0.35	0.93 (0.11 – 8.14)	0.95	0.80 (0.31 – 2.08)	0.64
**Donor TelB and type C2 ligand graft-versus-host mismatch**	<0.01 (<0.01)	<0.001	<0.01 (<0.01)	<0.001	1.14 (0.11 – 11.57)	0.91	0.53 (0.08 – 3.66)	0.52	3.61 (1.07 – 12.20)	0.04

Multivariate analysis of overall survival was conducted with Cox regression. In multivariate analyses of the other types of events (relapse, relapse-related mortality, non-relapse mortality, and neutrophil engraftment), we corrected for competing risks using the Fine-Gray model. Hazard ratios less than 0.01 are uniformly shown as "<0.01", and 95% C.I.'s whose upper limits are <0.01 are shown as "<0.01". P values less than 0.001 are shown as "<0.001". All the reported P values were not adjusted for multiple-hypothesis testing.

[*] All the cases in the study cohort were MAC.

Figure S1. Flow chart of UCBT patient selection.

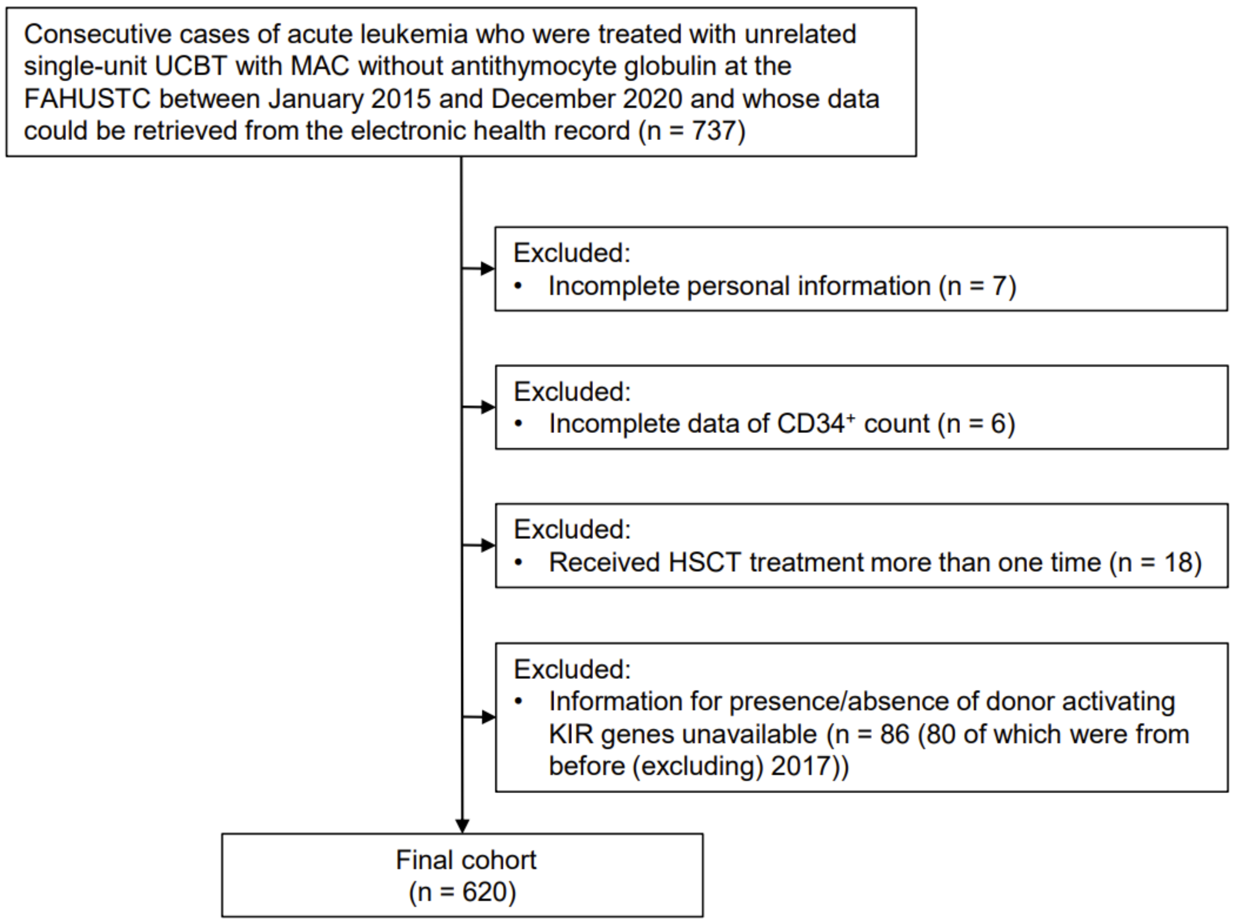

Figure S2. Distribution of infused CD34^{+} cell dosage in the UCBT cases.

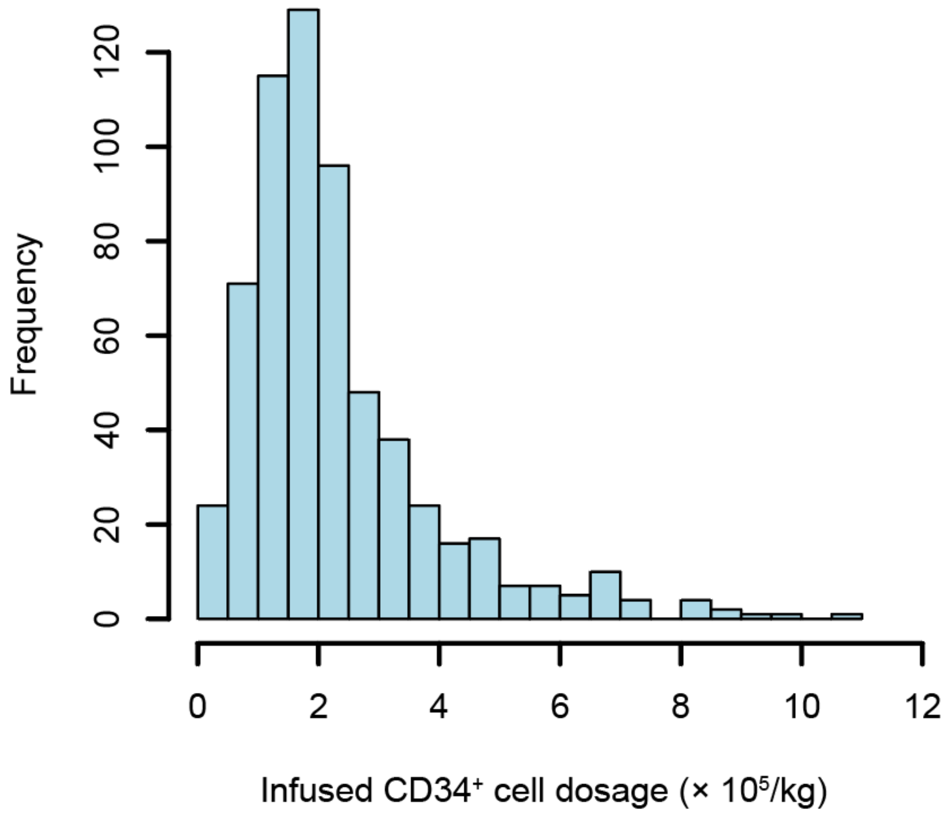

Figure S3. Flow chart of MSD HSCT patient selection.

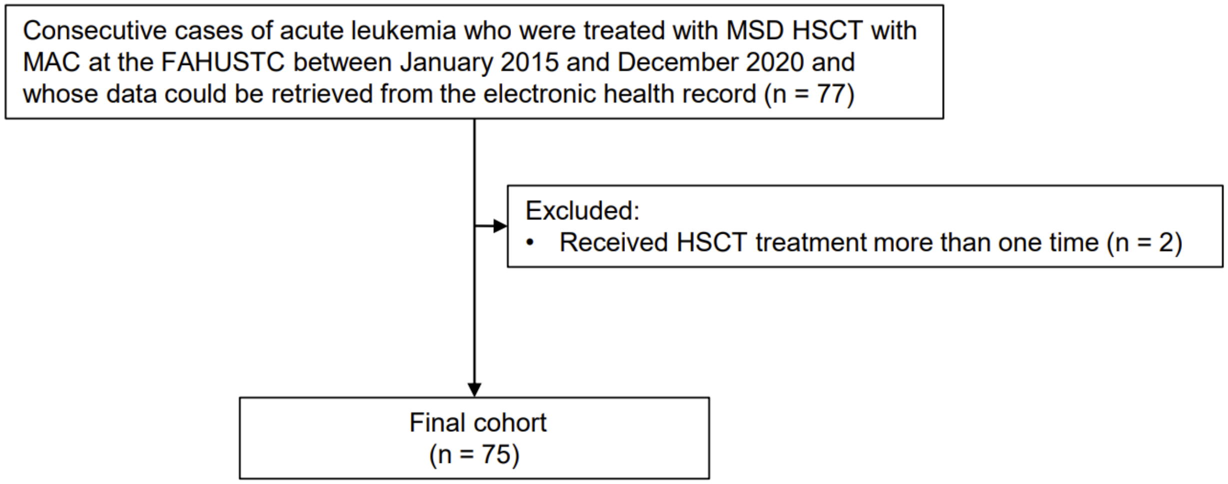

Figure S4. Linkage disequilibrium structure of donor KIR genes in the UCBT cases.

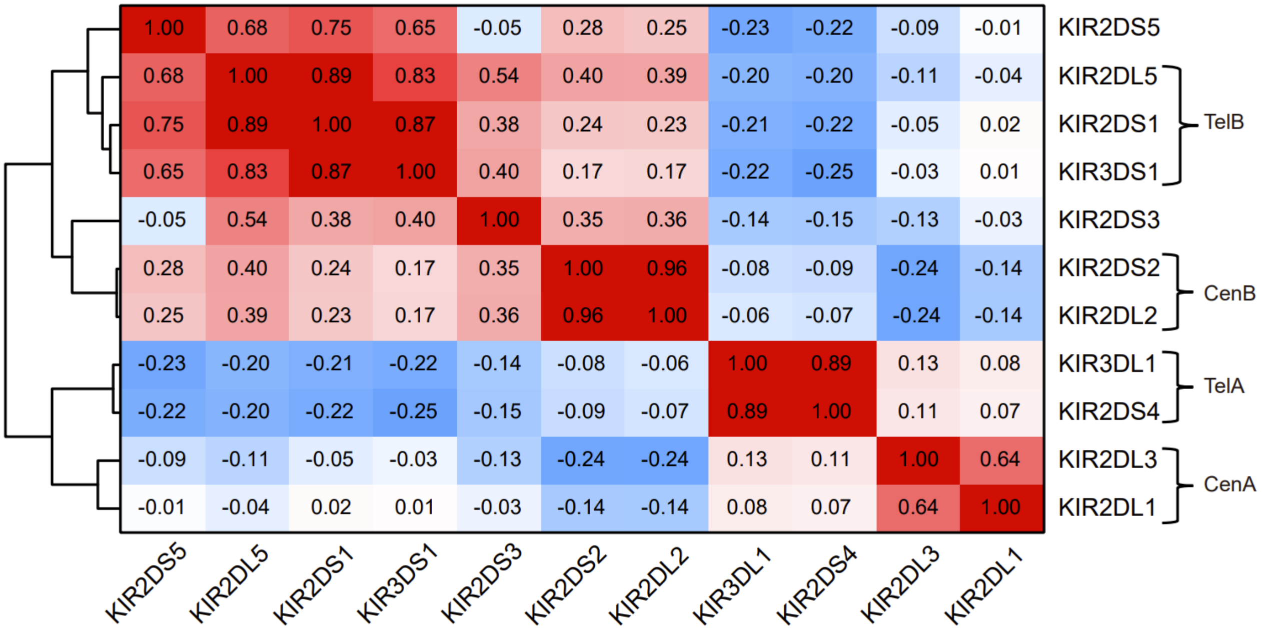

Shown values are pairwise Pearson's correlation coefficients (range –1 to 1) of the presence/absence of genes. Hierarchical clustering was performed using complete linkage with Euclidean distances on the pairwise correlation coefficient matrix. Framework genes (KIR2DL4, KIR3DL2, KIR3DL3, and KIR3DP1) were excluded from this analysis.
